# Increases in the Risk of Cognitive Impairment and Alterations of Cerebral β-amyloid Metabolism in Mouse Model of Heart Failure

**DOI:** 10.1371/journal.pone.0063829

**Published:** 2013-05-30

**Authors:** Xiaoqi Hong, Liping Bu, Yi Wang, Jing Xu, Jian Wu, Yufang Huang, Jie Liu, Haiyun Suo, Lumeng Yang, Yuncen Shi, Yi Lou, Zhengliang Sun, Guoqi Zhu, Thomas Behnisch, Mei Yu, Jianguo Jia, Wangxi Hai, Hongping Meng, Sheng Liang, Fang Huang, Yunzeng Zou, Junbo Ge

**Affiliations:** 1 State Key Laboratory of Medical Neurobiology, Shanghai Medical College and Institutes of Brain Science, Fudan University, Shanghai, China; 2 Department of Cardiology and Shanghai Institute of Cardiovascular Diseases, Zhongshan Hospital, Fudan University, Shanghai, China; 3 Institutes of Biomedical Sciences, Fudan University, Shanghai, China; 4 Key Laboratory of Smart Drug Delivery, Ministry of Education and PLA, Fudan University, Shanghai, China; 5 Med-X Research Institute, Shanghai Jiao Tong University, Shanghai, China; 6 Department of Nuclear Medicine, Ruijin Hospital, School of Medicine, Shanghai Jiaotong University, Shanghai, China; Cleveland Clnic Foundation, United States of America

## Abstract

Epidemiological and clinico-pathological studies indicate a causal relationship between heart disease and Alzheimer’s disease (AD). To learn whether heart disease causes an onset of AD, mice with myocardial infarction (MI) and congestive heart failure (HF) were used to test neuropsychiatric and cognitive behaviors as well as for measurements of AD related protein markers. To this end, adult mice were subjected to ligation of left anterior descending artery (LAD) and about two weeks later high-frequency echocardiography was performed to exam the resulting cardiac structure and function. Three months after successful induction of chronic heart failure (CHF) these mice showed an impairment of learning in the Morris Water Maze task. In addition, the expression of selected molecules, which are involved in β-amyloid metabolism, apoptosis and inflammation on the level of gene transcription and translation, was altered in CHF mice. Our findings provide a plausible explanation that CHF increases the risk of cognitive impairments and alters cerebral β-amyloid metabolism. In addition, our data indicate that the cerebral compensatory mechanisms in response to CHF are brain area and gender specific.

## Introduction

Heart disease and Alzheimer’s disease (AD) are main threats to public health. Epidemiology and clinico-pathology show that heart disease may be causal in the progress of AD [1]–[4]. Indeed, it was found that heart disease and AD share many pathological mechanisms, such as amyloid oligomers, alterations of mitochondrial DNA, oxidative stress and inflammation [5]–[8]. Heart failure is one of the most prevalent forms of heart diseases [9], leading to a decreased cerebral blood flow (CBF) in human [2], [3], [10], [11]. For example, patients with severe heart failure had a CBF reduction of approximately 30% [12]. In addition, neuroimaging data support that AD-like brain changes may develop in heart failure patients, possibly as a consequence of chronic CBF reductions [13]. Such a lasting reduction in the supply of oxygen, glucose and other nutrients to the brain causes neuronal cell death in many brain areas, including the hippocampus and posteriorparietal cortex [8], [13], [14]. These brain areas are known for their involvement in distinct forms of learning and memory and other cognitive abilities [8], [15]. Indeed, the prevalence of cognitive dysfunction, such as impairment of executive function, memory, language, mental speed and attention, in patients with congestive heart failure was estimated to be 25 to more than 50% [10], [16]–[18]. In addition, a pharmaco-epidemiological survey indicated that digoxin, a drug used to treat congestive heart failure and atrial fibrillation improves cognitive performance among senile patients with heart failure [19]. Although, there are several indications suggesting a tight connection between heart disease and declining cognitive abilities in human [20], [21], a mouse model to study such relationship as well as the effect of heart failure on amyloid metabolism, a protein marker for AD is so far missing.

To this end, we induced myocardial infarction/congestive heart failure in mice by occlusion of the left anterior descending coronary artery followed by high-frequency echocardiography to exam the cardiac structure and function two weeks later. Locomotor activity and in addition neuropsychiatric and cognitive behaviors of such mice were evaluated within several weeks after surgery. Three months later, the metabolic pathways of β-amyloid in the hippocampus and cortex were assessed by quantitative RT-PCR and Western blot analysis and the level of amyloidogenesis, neuronal degeneration and glial activation determined. Furthermore, mice with a three or six-month course of heart disease were subjected to **^1^** ^8^F-fluoro-deoxyglucose ([^18^F]-FDG) MicroPET/CT imaging assays to analyze glucose uptake in the brain and heart. Our data provide evidence for the induction of gender specific risks of cognitive impairments and AD related pathology by CHF.

## Materials and Methods

### Animal

C57BL/6J mice were purchased from Shanghai Branch of National Rodent Laboratory Animal Resources. Mice were housed at 24°C under 12 h light/dark cycles with free access to food and water. All mouse care and experimentation were approved by the Institutional Animal Care and Use Committee of Fudan University Shanghai Medical College (IACUC Animal Project Number: 20080307-055). All surgery was performed under chloral hydrate anesthesia, and all efforts were made to minimize suffering.

### Induction of Myocardial Infarction/congestive Heart Failure

Mice (10–14 weeks old) were subjected to left anterior descending coronary artery **(**LAD) ligation to induce myocardial infarction (MI)/congestive heart failure as previously reported [22]. Briefly, mice were anesthetized by intraperitoneal injection of a mixture of ketamine (150 mg/kg) and xylazine (10 mg/kg), then endotracheally intubated and cannulated to a rodent ventilator (Type 7025, Harvard Apparatus, March-Hugstetten, Germany). Parasternal thoracotomy was performed in the third intercostal space; LAD coronary artery was ligated with a 7–0 polypropylene suture around 1 mm distal to the left auricle under a microscope (Nikon SM2645, Tokyo, Japan). All of the mice in the MI group presented myocardium blanching according to direct visualization. SHAM-operated mice with thoracotomy but without LAD ligation were served as the control.

### Echocardiography

Echocardiography was performed using micro-ultrasound imaging system (Vevo770, VisualSonics Inc., Toronto, Canada) with a 30-MHz center frequency scanhead about two weeks after surgery. Positioned on a heating pad to maintain normothermia, mouse was anesthetized using isoflurane (4% for anesthesia induction and 1% for maintenance). Ventricular structure and systolic function were measured as we previously described [23]. Parameters included left ventricular end-diastolic and end-systolic dimensions (LVEDD and LVESD), left ventricular anterior wall end-diastolic and end-systolic thickness (LVAWTd and LVAWTs), left ventricular posterior wall end-diastolic and end-systolic thickness (LVPWTd and LVPWTs), left ventricular ejection fraction (LVEF) and fractional shortening (LVFS) and LVMass [24]. Heart failure was defined based on the in vivo LV dysfunction (LVEF <40% in this study).

### Behavioral Assessment of the Experimental Mice

#### Open field test

Basal locomotor activity of mice was evaluated with an automatic-recording open-field working station (MED Associates, Georgia, VT, USA) by analyzing the total walking (traveling) distance and the duration of ambulatory and stereotypic movements as well as resting over a time of 30 minutes [25].

#### Elevated plus-maze test

The elevated plus-maze (EPM) test was performed to test anxiety-like behaviors as described by Simonin et al [26]. Each mouse was placed in the central part of the cross maze (Med Associates, Inc., St. Albans, Vermont, USA), and allowed to move freely for 5 minutes. Time spent in each arm and exploration and entry frequency were calculated.

#### Morris water maze

The Morris water maze test was performed as described previously and used to characterize the spatial learning performance of SHAM-operated and ligated mice [27], [28]. Mice were allowed up to 60 seconds to locate the escape platform. If an animal failed to find the platform within the period then it was gently guided to the platform and allowed to stay on it for 30 seconds. Each mouse performed four trials daily for six consecutive days from randomly chosen starting points. 24 hours after the last trial, a single probe trial was performed.

### PET/CT Imaging Protocols, Image Reconstruction and Quantitative Evaluation

To determine the volume of heart and brain and their levels of glucose uptake and metabolism FDG-MicroPET/CT scans were conducted. PET/CT imaging was performed on an Inveon MM Platform (Siemens Preclinical Solutions, Knoxville, Tennessee, USA) with a computer-controlled bed and 8.5 cm transaxial and 5.7 cm axial fields of view. The animals were anesthetized with 2% isoflurane in O_2_ gas for [^18^F]-FDG injection (a single injection of 0.1 ml FDG with an activity of about 10MBq via the tail vein). 40 minutes after the tracer injection, animals were placed prone on the PET scanner bed near the central field of view and were maintained under continuous anesthesia during the study with 1.5% isoflurane in oxygen at 2 L/min. Inveon Acquisition Workplace (IAW) 1.4.3.6 was used for scanning process. 10 min CT X-ray for attenuation correction was scanned with a power of 80 Kv and 500 µA, and an exposure time of 1100 ms before PET scan. Ten-minute static PET scans were then acquired, and images were reconstructed by an OSEM3D (Three-Dimensional Ordered Subsets Expectation Maximum) algorithm followed by MAP (Maximization/Maximum a Posteriori) or FastMAP provided by IAW. The 3D regions of interest (ROIs) were drawn over the heart or brain guided by CT images and tracer uptake was measured using the software of Inveon Research Workplace (IRW) 3.0. Individual quantification of the [^18^F]-FDG uptake was calculated. Mean standardized uptake values (SUV) were determined by dividing the relevant ROI concentration by the ratio of the injected activity to the body weight.

### Evaluation of Blood Brain Barrier Integrity

Quantitative Evans blue (EB) analysis was carried out as previously described [29]. Briefly, mice were intravenously injected with 2% EB saline solution via tail vein. 2 hours later, mice were perfused with saline until the drainage was colorless. After decapitation, the brain was removed, weighed and immersed into formamide (1 ml/100 mg) at 55°C for 72 h and then centrifuged. The optical density of supernatants was determined at 611 nm with a spectrophotometer (Biorad, Hercules, CA, USA). The tissue content of EB was quantified from a linear standard curve and was expressed as micrograms per gram of brain tissue.

### Real-time PCR

Real-time PCR was performed for quantification of *APP*, *BACE1*, *Bcl-2*, *Bax*, *IL-1β*, *IL-6*, *iNOS*, *TLR4*, and *TNF-α* expression on a quantitative thermal cycler (Mastercycler ep realplex, eppendorf, Germany). The primers used in the real-time PCR were as follows:

β-actin forward: 5′ATGAGGTAGTCTGTCAGGT3′

β-actin reverse: 5′ATGGATGACGATATCGCT3′

BACE1 forward: 5′GCATCGCTACTACCAGAGGCA3′

BACE1 reverse: 5′GGTCTGCTTCACCAGGGAGTC3′

APP forward: 5′TGCTGGCAGAACCCCAGATCG3′

APP reverse: 5′TTCTGGATGGTCACTGGCTGG3′

Bax forward: 5′GCGTGGTTGCCCTCTTCTACTTTGC3′

Bax reverse: 5′GAAGAAAAGACACAGTCCAAGGCAG3′

Bcl-2 forward: 5′GGATTGTGGCCTTCTTTGAGTTCGG3′

Bcl-2 reverse: 5′CATATTTGTTTGGGGCAGGTTTGTC3′

IL-6 forward: 5′CAACGATGATGCACTTGCAGAAAAC3′

IL-6 reverse: 5′TCTGTGACTCCAGCTTATCTGTTAG3′

iNOS forward: 5′TGGCCACCTTGTTCAGCTACG3′

iNOS reverse: 5′TGAGTTCGTCCCCTTCTCCTGTTGG3′

TLR4 forward: 5′CTAGGACTCTGATCATGGCAC3′

TLR4 reverse: 5′AATCCAGCCACTGAAGTTCTG3′

TNF-α forward: 5′CACGCTCTTCTGTCTACTGAACTTC3′

TNF-α reverse: 5′GCAGCCTTGTCCCTTGAAGAGAACC3′

IL-1β forward: 5′GCAACTGTTCCTGAACTC3′

IL-1β reverse: 5′CTCGGAGCCTGTAGTGCA3′

### Protein Extraction and Western Blot Analysis

The method of protein extraction and Western blot analysis has been described elsewhere [30]. The following primary antibodies were used: rabbit anti-APP (A8717, 1∶5000; Sigma, St. Louis, USA ), mouse anti-t-Tau (Tau-5, 1∶2000; Millipore, Billerica, MA, USA), rabbit anti-BACE1 (Anti-B690, 1∶1000; Calbiochem, USA), rabbit anti-p-Tau (pSer202, 1∶2000; LifeSpan BioSciences, USA), rabbit anti-Bax and rabbit anti-Bcl-2 (#2772 and #2876, 1∶1000; Cell Signaling Technology, USA), and mouse anti-β-actin (SC-47778, 1∶1000; Santa Cruz Biotechnology, USA). The protein levels were quantified by densitometry analysis using Quantity One 4.5.2 software (Bio-Rad, Hercules, CA, USA).

### Thioflavin S and Fluoro-jade B Staining

Brain slices were placed in 1% Thioflavin S for 5 minutes, differentiated in 70% alcohol for 5 minutes. The slices were then rinsed in distilled water two times, mounted in glycerin jelly. Amyloid aggregates were detected under the fluorescence microscope (Olympus, Tokyo, Japan).

Fluoro-Jade B (FJB) staining procedure was performed to examine degenerating neurons as described elsewhere [31]. Briefly, the slides were immersed in the 0.004% FJB staining solution for 20 min, rinsed for one minute in distilled water three times. FJB signals were detected at an excitation of 480 nm and an emission of 525 nm under an epifluorescence microscope (Olympus, Tokyo, Japan).

### Immunofluorescence Staining

Brain sections were permeabilized and blocked in PBS containing 0.2% Triton X-100 and 10% normal goat serum 37°C for 1 hour, and then stained with mouse monoclonal anti-neuronal nuclei (NeuN) (MAB377, 1∶200; CHEMICON, USA) and rabbit polyclonal anti-GFAP (AB5804, 1∶1000; CHEMICON, USA) or rabbit anti-Iba1 (019-19741, 1∶500; Wako, Japan). The sections were incubated with the primary antibodies at 37°C for 2 hours, and overnight at 4°C. After washing with PBS, the sections were incubated with anti-mouse IgG-Alexa Fluor488 (A11029, 1∶1000; Invitrogen, USA) and anti-rabbit IgG-Alexa Fluor647 (A31573, 1∶1000; Invitrogen, USA) for 1 hour at 37°C. Images were obtained using a Leica DM LB2 microscope (Leica, Wetzlar, Germany)**.**

### Statistical Analysis

Data were analyzed using SPSS software (version 17; SPSS, Chicago, USA). All values were expressed as means ± SE. Statistical analysis was determined using Student’s t test (when two groups were considered) or by one-way analysis of variance (ANOVA) followed by multiple comparisons with the LSD *post-hoc* test. *P*<0.05 was considered significant.

## Results

### Evaluation of Surgically Induced Heart Failure

**Figure 1:**
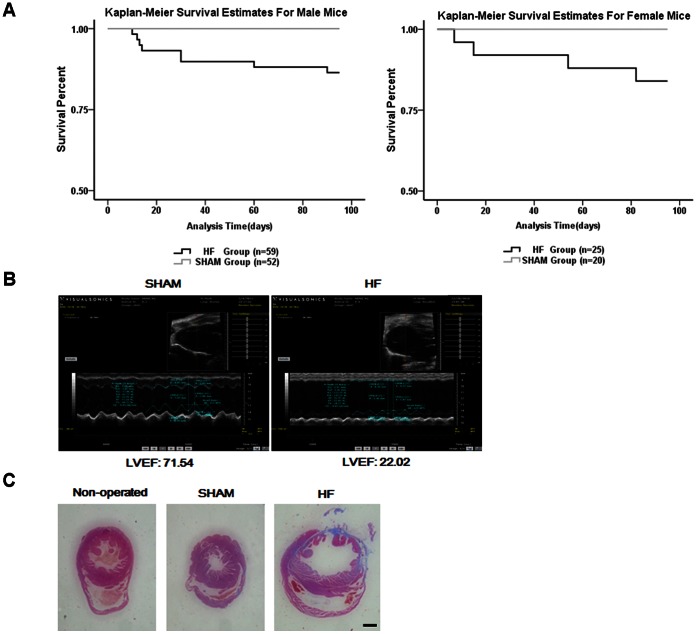
The survival estimates, and cardiac structure and function of HF and SHAM mice. (A) The survival estimates surveyed during the experimental procedures were shown. (B) Representative M mode echocardiograms of HF and SHAM mice were shown. (C) Representative hematoxylin and eosin (HE) staining of heart slices from control and HF mice three months after surgery was shown. Scale bar: 1 mm.

To test if SHAM or LAD surgeries have an impact on mouse survival, we did the survival estimates. Three months after surgery, the survival rate for male and female mice with heart failure was 86.4% and 84%, respectively (Figure 1A). The effect of such surgeries on feeding behavior and metabolism was also analyzed. In response to surgeries the body weight of male mice of the SHAM and HF group was not distinguishable at time points applied for behavioral and biochemical investigations. However, the body weight of female mice with LAD ligation was significantly increased in comparison to SHAM mice at 4 to 12 weeks post-surgery, but not at 13th week (Figure S1).

Moreover, to specify whether LAD surgeries were sufficient to cause heart failure echocardiographic analyses of cardiac parameters for each mouse of both groups were conducted two weeks post-surgery. Representative echocardiographic images from SHAM and HF mice were shown in Figure 1B. The acquired echocardiographic parameters are listed in Table 1 and especially the LVEF is the reliable index of cardiac function [1], [32]. A LVEF value lower than 40% was taken as the threshold value to define whether surgery caused heart failure or not. The average LVEF of mice with cardiac surgery was 16.9% and 18.7% in males and females, respectively, in our experiments (Table 1).

**Table 1:** Echocardiographic comparison of HF and SHAM mice.

	Male		Female	
	SHAM (n = 11)	HF (n = 13)	SHAM (n = 9)	HF (n = 16)
LVEDD (mm)	3.96±0.08	5.72±0.29**	3.64±0.14	5.37±0.15**
VPWTd (mm)	0.69±0.02	0.6±0.06	0.65±0.02	0.7±0.04
LVESD (mm)	2.62±0.11	5.34±0.33**	2.41±0.19	4.92±0.17**
LVPWTs (mm)	1.07±0.05	0.74±0.07**	0.95±0.04	0.92±0.06
LVAWTd (mm)	0.72±0.02	0.35±0.04**	0.73±0.03	0.35±0.03**
LVAWTs (mm)	1.08±0.03	0.41±0.07**	1.14±0.03	0.42±0.05**
LVVol;d	68.7±3.31	168.19±19.94**	56.8±5.06	141.74±8.6**
LVVol;s	25.86±2.76	146.77±20.08**	22.2±3.55	116.87±8.75**
EF (%)	62.91±2.75	16.9±2.7**	63.19±4.1	18.7±2.11**
FS (%)	33.99±2.05	7.8±1.29**	34.49±3.38	8.63±1.02**
LVMass (mg)	97.7±4.4	108.03±8.17	82.77±4.99	117.89±7.72**

LVEDD and LVESD, left ventricular end-diastolic and end-systolic dimensions; LVAWTd and LVAWTs, left ventricular anterior wall end-diastolic and end-systolic thickness; LVPWTd and LVPWTs, left ventricular posterior wall end-diastolic and end-systolic thickness; LVEF, left ventricular ejection fraction; LVFS, left ventricular fractional shortening; LVMass, left ventricular mass. Values are means ± SE,

** *P*<0.01.

In addition, the anatomy of the heart was visualized by hematoxylin and eosin staining. Mice with LAD ligation were characterized by enlarged left ventricles and obvious fibrosis in comparison with hearts of wild-type or SHAM-operated mice (Figure 1C). Therefore, our surgical procedure was effective and sufficient to cause cardiac dysfunction and CHF in mice.

### Glucose Metabolism in the Heart and Brain, and Blood Brain Barrier Integrity in Mice with Chronic Heart Failure

**Figure 2:**
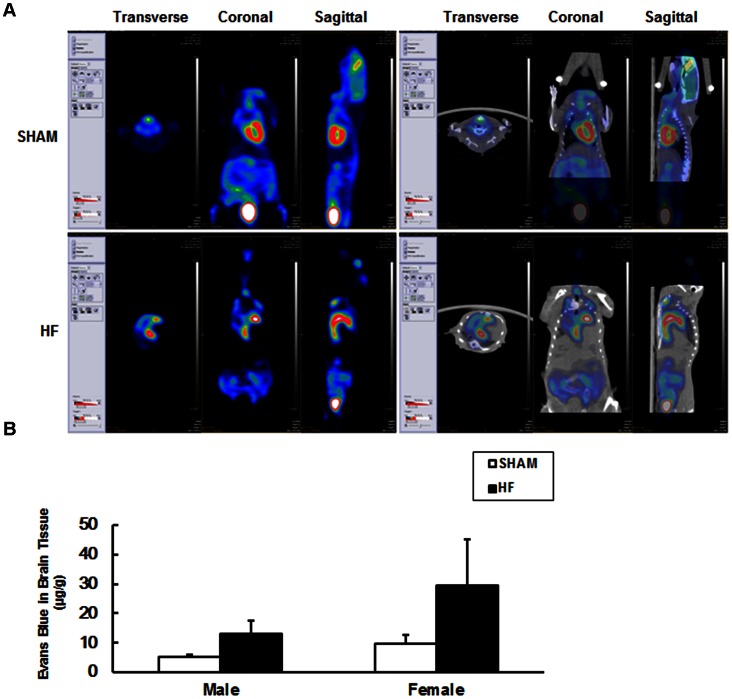
MicroPET/CT imaging and the integrity of blood brain barrier (BBB) in CHF and SHAM mice. (A) Three months after surgery, male CHF and SHAM mice were subjected to MicroPET/CT analysis. Representative MicroPET/CT images showing the glucose uptake and metabolism in the heart and brain were displayed. (B) The permeability of blood brain barrier (BBB) was indicated by extravasations of Evans blue dye. Evans blue concentrations (µg/g) in the brains of CHF and SHAM mice were assessed three months after surgery. Data are expressed as mean ± SE, n = 3–5 for SHAM and n = 4 for CHF group.

By FDG-MicroPET/CT, a variety of parameters, including the volume of heart and brain and their levels of glucose uptake and metabolism were determined. Three months after surgery, the average heart volume of male mice with CHF was two times as large as that of SHAM group, but the brain volumes of these mice were not distinguishable. Comparison of the glucose uptake in hearts from male mice of the SHAM or CHF group revealed that the mean and minimum SUV of ^18^F-FDG in the CHF group were less than half of those of SHAM mice. However, the mean, minimum and maximum SUV of brains from both groups were not distinguishable (Figure 2A and Table 2). The effects of CHF on glucose metabolism in the heart and brain of female mice were also characterized by MicroPET/CT six months after surgery. We observed that the minimum SUV of ^18^F-FDG in the hearts of female CHF mice was less than half of the value of SHAM mice, while the mean, minimum and maximum SUVs in brains manifested no difference similar to male CHF mice (Table 3 and Figure S2).

**Table 2:** The heart and brain volumes, glucose uptake/metabolism in the hearts and brains of male CHF and SHAM mice.

	Heart				Brain			
		SUV				SUV		
	Volume (mm^∧^3)	Mean ± SD	Min ± SD	Max ± SD	Volume (mm^∧^3)	Mean ± SD	Min ± SD	Max ± SD
SHAM (n = 3)	317.83±96.02	8.03±2.14	2.1±0.75	14.93±3.88	250.37±23.47	2.5±1.14	1.12±0.59	3.63±1.55
HF (n = 4)	633.23±165.65*	3.6±1.33*	0.82±0.17*	10.1±3.35	237.62±64.77	3.52±0.61	1.47±0.36	6±1.36

Male mice were subjected to MicroPET/CT scanning three months after surgery. Values are mean ± SD; Min: minimum; Max: maximum, *P<0.05.

**Table 3:** The heart and brain volumes, glucose uptake/metabolism in the hearts and brains of female CHF and SHAM mice.

	Heart				Brain			
		SUV				SUV		
	Volume (mm^∧^3)	Mean ± SD	Min ± SD	Max ± SD	Volume (mm^∧^3)	Mean ± SD	Min ± SD	Max ± SD
SHAM (n = 6)	213.95±65.85	6.77±3.5	2.68±0.96	11.18±6.61	218.1±18.17	2.87±0.92	1.57±0.65	4.17±1.25
HF (n = 3)	521.47±148.31**	5.27±0.93	1.02±0.5**	11.67±1.27	253.3±60.2	3.87±1.29	1.83±0.21	6.37±2.76

Female mice were subjected to MicroPET/CT scanning at six months after the heart surgery. Values are mean ± SD; Min: minimum; Max: maximum, **P<0.01.

In a next step, the level of blood brain barrier (BBB) integrity was studied using Evans Blue dye (EB). EB is known to bind to serum albumin and has been used as a tracer for assessing BBB disruption [29]. We found the CHF increased the cerebral concentration of the dye in both male and female mice slightly, however, non-significantly three months after surgery (Figure 2B).

### Strength of Locomotion, Anxiety and Learning is Altered in Mice with Heart Failure

**Figure 3:**
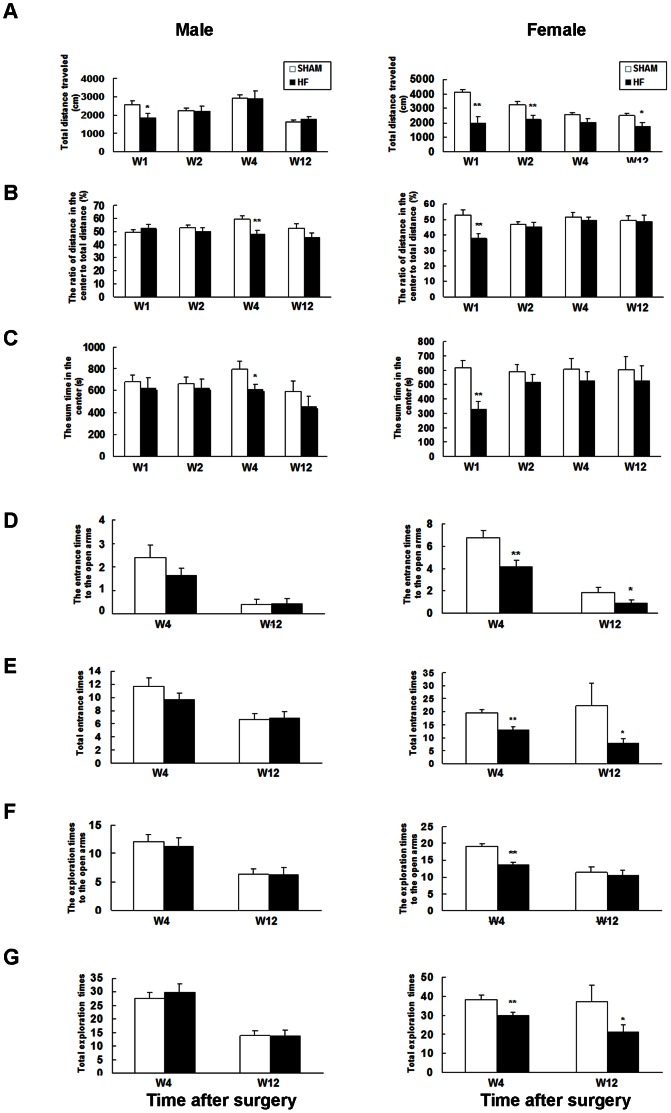
The results from open field test (A, B, C) and elevated plus-maze test (D, E, F, G). The bar diagrams show: (A) Total distance traveled (cm); (B) The ratio of distance traveled in center to total distance (%) and (C) The sum time spent in the center in the open field test. (D) The entrance times to the open arms; (E) The number of total entrances; (F) The number of explorations into the open arms and (G) Total explorations into the four arms in the elevated plus-maze test. Mice were tested at different time after surgery. Data are expressed as mean ± SE, *P<0.05, **P<0.01, n = 8–10 for SHAM and n = 10–12 for HF group in the open field test and the elevated plus-maze test.

The open field test (OFT) is often used to assess locomotor activity and anxiety-like behavior. In this study, OFT was performed one, two, four and twelve weeks (w1, w2, w4 and w12, respectively) after surgery. The total distance traveled in the open field was reduced significantly at w1, w2 and w12 in female mice with heart failure. Interestingly, for male heart failure mice, a similar shortening of the travel distance in comparison to SHAM group was only detectable one week after surgery (Figure 3A). The ratio of distance traveled in the center to total distance and the sum time in the center was significantly decreased at w1 and w4 in female and male mice with heart failure compared to SHAM mice, respectively (Figure 3B, C).

A decreased locomotion in female heart failure mice was also confirmed in elevated plus maze test (EPM), as the total entry times and entry times to the open arms decreased significantly (Figure 3D, E). In addition, the total exploration times decreased at w4 and w12 after surgery, the exploration times to the open arms were also reduced at w4 in female mice with heart failure (Figure 3F, G). Compared with female mice, male mice with HF displayed no differences in EPM assays. Interestingly, male HF mice were less sedative in tail suspension test indicated by the considerably decreased time below the lower threshold (Figure S3).

**Figure 4:**
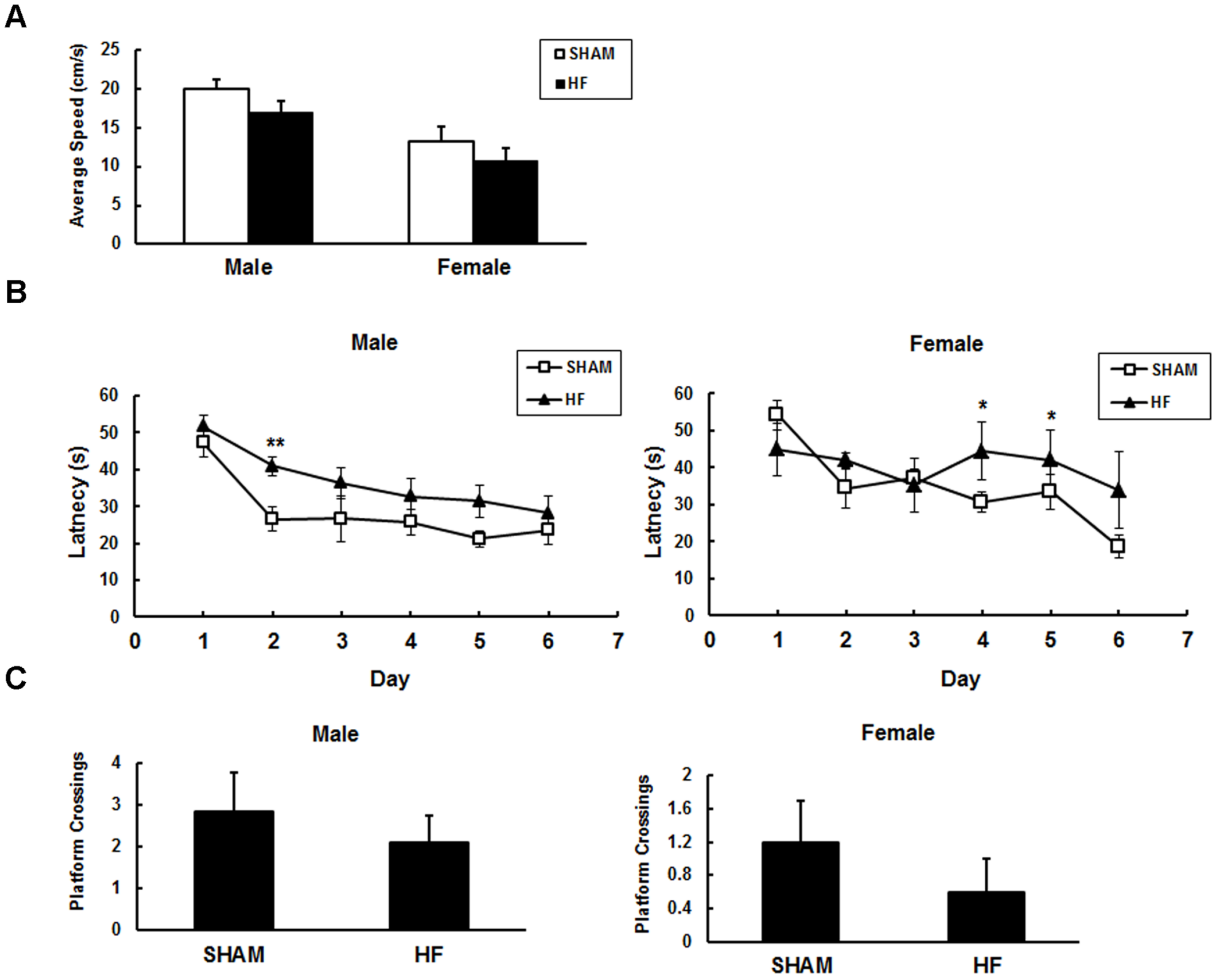
The effects of chronic heart failure on spatial learning and memory in mice. Cognitive behavioral tests were performed by the hidden-platform Morris water maze test. (A) There were no significant changes in the mean swimming speed for each group (P>0.05); (B) The average (4 trails per day) escape latency in water maze training was measured for 6 days. (C) Crossing times over the former platform location were shown. Twenty-four hours after the acquisition phase, mice were subjected to a probe test in which the platform was removed. Mice were tested three months after surgery. Data are expressed as mean ± SE, *P<0.05, **P<0.01, n = 5–7 for SHAM and n = 5–11 for CHF group.

The Morris water maze has been utilized widely to assess spatial memory and learning. Both male and female mice with CHF showed similar swimming velocity as their counterparts three months after surgery (w12; Figure 4A). However, female CHF mice required a longer time to find the platform on the fourth and fifth day. Cardiac deficient male mice took more time to find the platform on the second day (Figure 4B). Both female and male mice exhibited no difference in the probe trial on the seventh day (Figure 4C). In addition, in the novel object recognition test, during the second 5 minutes, male CHF mice showed decreased preference to the novel object (Figure S4).

Moreover, in the electrophysiological experiment, we found that LTP in the region of hippocampus was normal in CHF mice three months after surgery (Figure S5).

### CHF Alters Transcriptions of Genes Involved in the Metabolism of β-amyloid, Inflammation and Apoptosis

**Figure 5:**
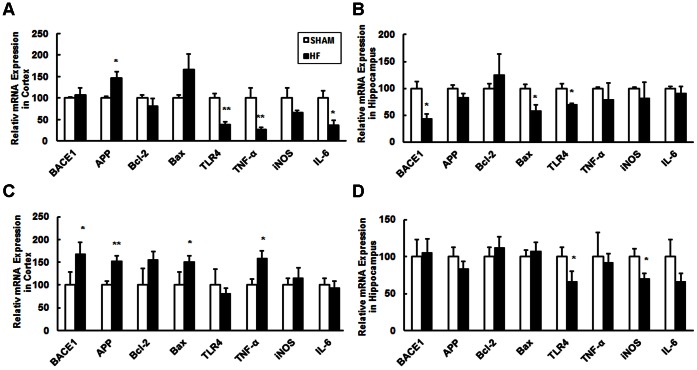
Transcriptional alterations of selected genes detected by real-time PCR in the brains of CHF mice three months after surgery. Transcriptional levels of selected genes in the male cortex (A) and hippocampus (B), female cortex (C) and hippocampus (D) were shown. All mRNA expression levels were normalized to β-actin. Data are presented as mean ± SE, *P<0.05, **P<0.01, n = 4–6 for SHAM and CHF group.

The genes selected in this study can be divided into three categories: the metabolism of β-amyloid, inflammation and apoptosis. We found that in the cortex of male CHF mice the expression level of *APP* increased and of *TLR4*, *TNF-α* and *IL-6* decreased three months after surgery (Figure 5A). In the same mice only the transcriptional expression of *BACE1*, *Bax* and *TLR4* in the hippocampus was found to be decreased (Figure 5B). In contrast, in female mice, CHF caused an increased expression of *BACE1, APP*, *TNF-α* and *Bax* in the cortex (Figure 5C) but a decreased expression of *TLR4* and *iNOS* in the hippocampus three months after surgery (Figure 5D). The cerebellum is a special region of high privilege against various kinds of stresses and indeed only reduced expression of *TLR4* was detected in the cerebellum of the same female CHF mice (Figure S6). The data here suggest that alterations in gene transcription in response to CHF are brain area and gender specific.

### CHF Alters Expression of Proteins Involved in the Pathology of AD

**Figure 6:**
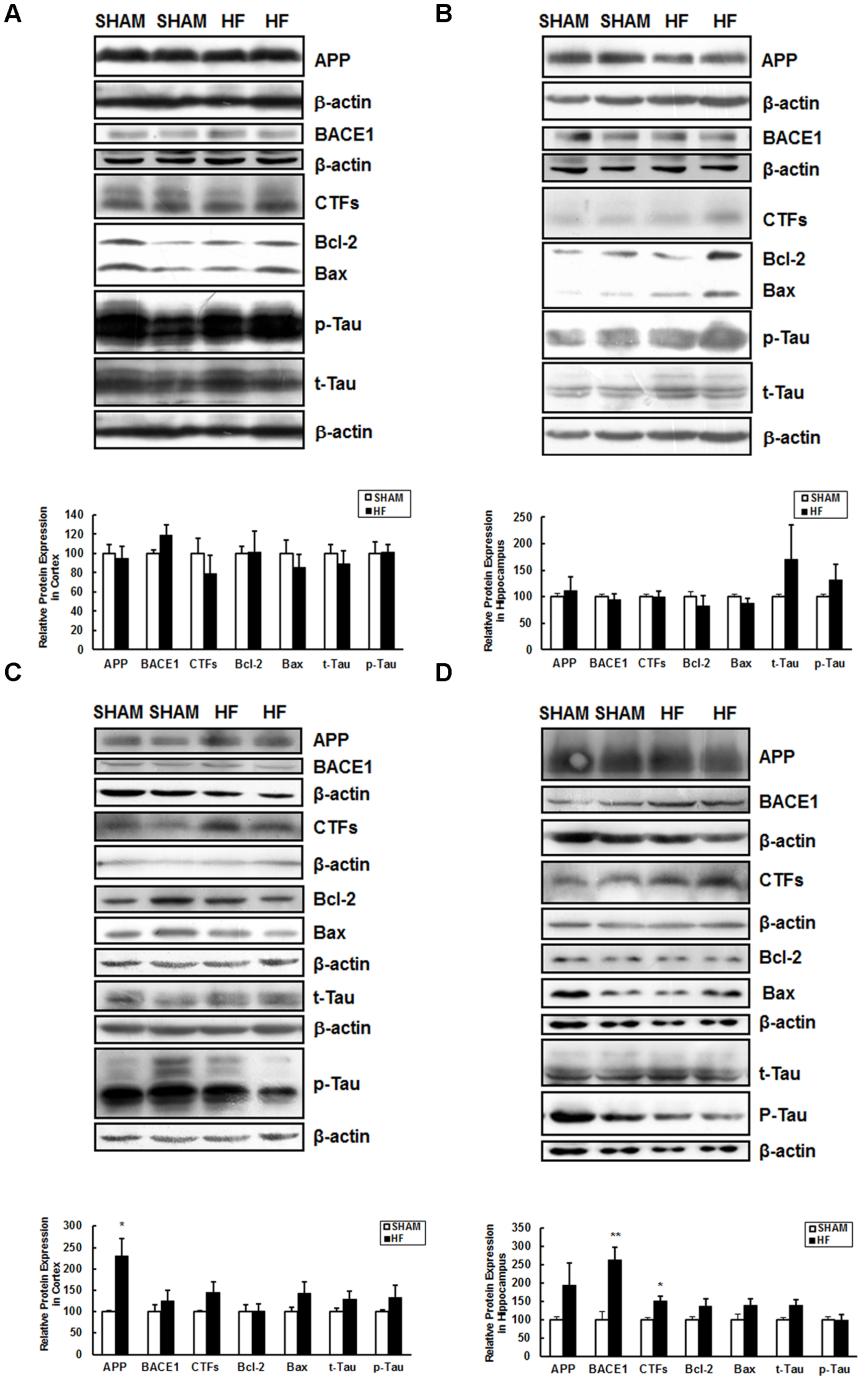
Expression levels of APP, BACE1, APP CTFs, Bcl-2, Bax, t-Tau, p-Tau proteins in the brain lysates of SHAM and CHF mice three months after surgery. Representative immunoblots and densitometric analysis of protein levels in the male cortex (A) and hippocampus (B), female cortex (C) and hippocampus (D) were depicted. β-actin served as the internal control. Data are presented as mean ± SE, *P<0.05, **P<0.01, n = 3–5 for SHAM and n = 4–6 for CHF group.

The effects of CHF on the expression of proteins involved in the pathology of AD were tested three months after surgery. Alzheimer’s disease is featured by extracellular β-amyloid accumulation and intracellular neurofibrillary tangles containing hyperphosphorylated Tau protein and progressive neuronal death. In the cortex and hippocampus of male CHF mice, the analyzed proteins, such as APP, BACE1, APP C-Terminal fragments (CTFs), Bax, Bcl-2, total Tau (t-Tau) and phosphorylated Tau (p-Tau) did not exhibited any alterations when compared with the respective protein levels in brains of the SHAM group (Figure 6A, B). While in the cortex of female mice, CHF elicited higher expression of APP; in the hippocampus, heart failure significantly increased the expression of BACE1 and CTFs. The protein levels of Bax, Bcl-2, total Tau and phosphorylated Tau exhibited no changes in these two brain areas (Figure 6C, D). In addition, the expression of APP, Bax, Bcl-2, total Tau and phosphorylated Tau in the cerebellum was not affected by CHF in female mice (Figure S7).

### CHF does not Aggravate β-amyloid Burden, Neuronal Degeneration and Glial Activation

**Figure 7:**
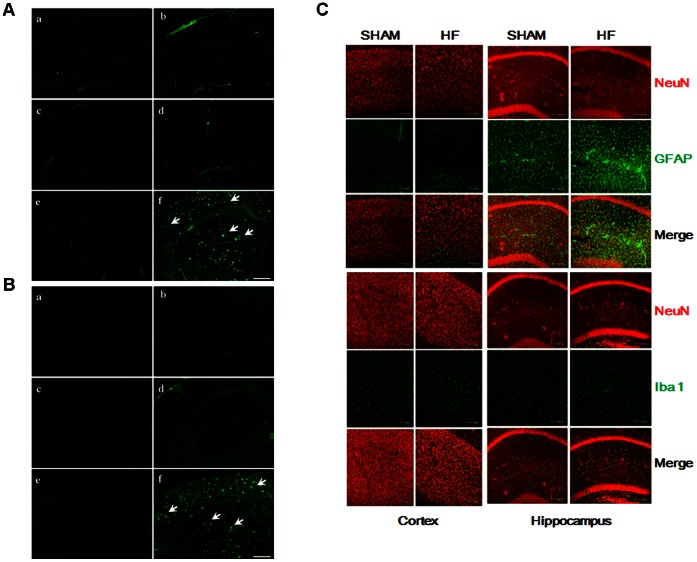
No amyloidogenesis, neuronal degeneration and activation of astrocytes and microglial cells on the brain slices of CHF mice. (A) Brain slices were stained with Thioflavin-S. Three months after surgery, Thioflavin S-stained amyloid plaques were not detected in the brain of male (a) SHAM and (b) CHF mice, female (c) SHAM and (d) CHF mice and (e) normal control mice, while the image in (f) depicts severe Thioflavin S-stained amyloid plaques (indicated by arrows) in the cortex and hippocampal area of APP/PS1 transgenic mice (12 months old). Scale bar: 500 µm. (B) Brain slices were stained with Fluoro-Jade B. Three months after surgery, no typical Fluoro-Jade B stained degenerating neurons were detected in the brain of male (a) SHAM and (b) CHF mice, female (c) SHAM and (d) CHF mice and (e) normal control mice, (f) APP/PS1 transgenic mice (12 months old). Arrows indicate typical spots of degenerating neurons. Scale bar: 500 µm. (C) Immunofluorescence staining of astrocytes and microglial cells in the brain slices of SHAM and CHF mice at six months post-surgery. Scale bar: 100 µm.

We investigated whether CHF causes accumulation of β-amyloid and affects neuronal survival in the cortex and hippocampus of male and female mice three months after surgery. Within the investigated brain structures of CHF mice positive signals were not detected, indicating that CHF does not induce amyloid accumulation and neuronal degeneration within the studied time window. However, brain slices from 12 month-old APP_swe_/PSEN1_ΔE9_ (APP/PS1) mice [33], known to posses amyloid plaques, were employed as a positive control to show the sensitivity of utilized detection methods. In these brain slices we found intense signals indicating amyloid accumulation and neuronal degeneration (Figure 7A, B).

To learn if GFAP expression is altered in mice three months after LAD ligation immunohistochemical stainings of GFAP positive astrocytes were analyzed and compared to stainings from SHAM mice. Our data indicate that there is no induction of additional astroglial GFAP expression in the hippocampus when compared to the SHAM group. Moreover, immunoblotting confirmed that LAD ligation does not induce additional expression of GFAP in the cortex and hippocampus when compared to respective GFAP levels in SHAM mice (Figure S8). A further attempt to find indications for CHF-induced gliosis, microglia activation or amyloidgenesis in mice 6 months after surgery failed. We found that astrocytes and microglial cells in the cortex and hippocampus of CHF mice were not distinguishable from the SHAM mice (Figure 7C). In addition, degeneration of neurons was not detected (data not shown) and β-amyloid accumulation was not observed based on Thioflavin-S staining (Figure S9).

## Discussion

Myocardial infarction (MI) is a leading cause of heart failure in human and it is often artificially induced in rodents by LAD ligation [34]–[36]. Whereas causal relationships between CHF and AD for human [21] have been described, animal studies are rare. Here we presented data indicating that CHF can increase a gender specific risk of developing cognitive dysfunction by modulation of amyloid processing and inflammation detected at the level of gene transcription and translation.

### Surgically Induced Heart Failure

To induce CHF in rodents ligation of LAD is widely used [34]–[36]. We found that hearts of mice after LAD surgery exhibited enlarged left ventricle and severe fibrosis within 3 to 6 months, which indicates pumping failure and decompensatory cardiac remodeling of end-stage heart disease. Similar cardiac hypertrophies are also common in human with CHF [37], [38]. Glucose hypometabolism in the hearts of CHF mice was demonstrated in the study (Figure 2A, Figure S2, Table 2, Table 3). A similar decrease of the glucose metabolism has been reliably described for human patients with CHF [39].

### Glucose Metabolism in the Brain

In healthy human brain, the level of cerebral blood flow is tightly coupled with local metabolic needs of brain areas in dependency upon cognitive activities. This statement is based on observations that within a vascular territory changes of CBF and glucose metabolic rate are almost linear [40]. We could not find any differences in cerebral glucose metabolism in CHF mice compared with SHAM mice by FDG-MicroPET (Figure 2A, Figure S2, Table 2, Table 3). We believe it is a reasonable outcome because the distribution of cardiac output is assumed to protect vital organ perfusion, such as the brain [12]. In fact, human resting cerebral blood flow is maintained quite constant within a wide range of mean arterial blood pressure. CBF is only reduced by approximately 30% in patients with New York Heart Association class III and IV CHF, while the value of LVEF is reduced by 67%–75% compared to healthy controls [12]. Therefore, there exist finely developed compensatory mechanisms to protect human brains from ischemic injury and most likely such mechanisms also work in mice.

A good agreement between hypoperfusion and hypometabolism was found in AD patients as indicated by altered CBF measured by magnetic resonance imaging (MRI) and FDG-PET, respectively [41]. In general AD and AD combined with intracerebral vascular disease account for 63%–73% of dementia cases [42]. In senile AD patients, a decreased glucose metabolism in temporo-parietal, posterior cingulate and medial temporal regions was consistently detected [43]–[45]. Glucose hypometabolism in patients with mild cognitive impairment (MCI) that was confined to cingulate and medial temporal cortices was reported by Furukawa et al [43], while Forsberg et al declared that there was no significant difference in the cerebral glucose metabolism between MCI patients and healthy controls [45]. The discrepancy in the two studies might be due to the age difference of MCI patients enrolled in these studies.

### Alterations in Expression of Selected Genes Involved in Inflammation and β-amyloid Metabolism

Chronic inflammation might be another link between CHF and declined cognitive abilities. Indeed, many studies prove that chronic inflammation plays a pivotal role in the progression of cardiovascular diseases and AD [46]. To learn if artificially induced CHF caused inflammation related gene expression we tested for *TLR4, TNF-α* and *IL-6* in the cortex and hippocampus and found significant alterations in male CHF mice. Interestingly, in female CHF mice the expression of inflammation related genes was different in comparison to male CHF mice. Thus, a decrease of heart function in an otherwise healthy body is altering the level of inflammation in the brain at the level of gene transcription.

One of the many hypotheses regarding molecular mechanisms of AD is the accumulation of amyloid proteins by mutation or upregulation of APP. We tested in this study weather CHF in mice is altering the level of APP and other AD related gene expression. In our experiment, the *APP* gene expression increased in the cortex of male mice under LAD surgery; only in female CHF mice, the levels of *APP* and *BACE1* mRNA were upregulated in the cortex. Besides, an increase of APP protein level in the cortex, and of BACE1 and APP CTFs in the hippocampus were observed. Increases in BACE1 expression and its enzymatic activity were also detected in the ischemic hippocampus of diabetic rat [47]. However, the modulation of these genes and proteins in CHF mice did not lead to detectable amyloid plaques or other biochemical hallmarks commonly studied in AD mice models or AD patients.

We found that the level of inflammation and amyloid metabolism is gender specific, which is in line with the results of multicenter studies which show that of the patients with CHF and systolic dysfunction, a large percentage has a diagnosis of Alzheimer’s disease and related disorders [21], and that women have a higher morbidity of dementia [20], [21].

### Locomotion, Anxiety and Learning in CHF Mice

The neuropsychological abnormalities, such as cognitive dysfunction and depression are common in CHF patients [11], [48]. We found CHF caused behavioral alterations in mice. In OFT, both male and female CHF mice showed to some extent lower locomotor activity, and more severe anxiety-like behavior (Figure 3 A, B, C). In the EPM paradigm, the exploration behavior decreased significantly in female mice with surgery (Figure 3F, G). Conversely, male mice with heart surgery were even more restless in tail suspension test (Figure S3). The preference to the novel object during the second 5 min was also reduced in male CHF mice (Figure S4). So, mice with CHF displayed to some extent changes in locomotor and neuropsychiatric behaviors. The impairment of spatial learning in MWM was quite moderate both in male and female CHF mice (Figure 4). Consistently, LTP in the region of hippocampus was indistinguishable between CHF and SHAM mice three months after surgery (Figure S5).

In our study, mice with severe CHF show moderate alterations in the brains, which somehow differs from human beings. Indeed, it was just recently published that inflammation response after burns, trauma and endotoxin in mice are different from responses seen in human [49].

In summary, we found that CHF and ensuing alterations of cerebral microenvironment lead to transcriptional and/or translational changes of β-amyloid and inflammation-related genes to a different extent in male and female mice as well as between cerebral cortex, hippocampus and cerebellum. Therefore, CHF itself can already increase the risk of cerebral inflammation, amyloid metabolism and cognitive impairments.

## Supporting Information

Figure S1 **Mouse weight surveyed during the experimental procedures.** There was no difference in the gain of weight in male mice of HF and SHAM groups; while the weight of female mice with surgery was significantly higher than that of SHAM group during W4 and W12, *P<0.05 or **P<0.01, n = 8–10 for SHAM and n = 10–12 for HF group.
(TIF)

Figure S2 **^18^F-FDG MicroPET/CT imaging exhibiting the glucose uptake and metabolism in the heart and brain of female SHAM and CHF mice at six months post-surgery.**
(TIF)

Figure S3 **The result from tail suspension test.** Tail suspension test was used to assess the depression-like behaviors of CHF and SHAM mice. The time of immobility and struggle was measured during a 6 minutes tail suspension test. Test was performed three months after surgery. Data are expressed as mean ± SE, *P<0.05, n = 5–10 for SHAM and n = 5–11 for CHF group.
(TIF)

Figure S4 **The result from novel object recognition test.** The novel object recognition task was used to evaluate the cognition ability in CHF and SHAM mice. Mice were exposed to two copies of an object for 10 minutes and then after 60 minutes they were allowed to explore the familiar object and a novel object again for 10 minutes. The object exploration time during the test phase was recorded. Test was performed three months after surgery. Data are expressed as mean ± SE,* P<0.05, n = 6–7 for SHAM and n = 4–5 for CHF group.
(TIF)

Figure S5 **Long term potentiation (LTP) was induced in hippocampal slices of CHF and SHAM mice three months after surgery.** (A) Schema of hippocampal slice and position of electrodes. The stimulation electrodes (S1, S2) were placed in the str. rad. of the hippocampal CA1 region facing each other. A recording electrode (gray, Rec) was placed in-between to record evoked fEPSPs. The S2 input was used as a control input to monitor baseline stability. Input S1 was used to evoke synaptic plasticity by high-frequency stimulation (HFS, 100 Hz). (B) LTP was induced by HFS, which consisted of 3 times 100 Hz tetanization (lasting 1 second) with an inter-train interval of 10 minutes. LTP was inducible in the acute slices obtained from CHF and SHAM mice and lasted over 90 minutes. No LTP impairments were detectable in CHF mice.
(TIF)

Figure S6 **Transcriptional alterations of selected genes detected by real-time PCR in the cerebellum of CHF mice three months after surgery.** All mRNA expression levels were normalized to β-actin. Data are presented as mean ± SE, *P<0.05, n = 4–6 for SHAM and CHF group.
(TIF)

Figure S7 **The expressions of APP, Bcl-2, Bax, t-Tau and p-Tau proteins in the cerebellum of SHAM and CHF mice three months after surgery.** Representative immunoblots and the summary of the densitometric analysis were shown. Data are presented as mean ± SE, n = 4–6 for SHAM and CHF group.
(TIF)

Figure S8 **Gliosis was not detectable within the cortex and hippocampus of CHF mice three months after surgery.** (A) Immunohistochemistry staining revealed GFAP positive astrocytes within the hippocampus of male (a) SHAM and (b) CHF mice, female (c) SHAM and (d) CHF mice. Scale bar: 20 µm. (B) The levels of GFAP proteins in the cortex and hippocampus of SHAM and CHF mice were determined by Western blot, n = 4–6 for SHAM and CHF group.
(TIF)

Figure S9 **No amyloidogenesis on brain slices of female CHF mice six months after surgery.** Brain slices were stained with Thioflavin-S. Thioflavin S-stained amyloid plaques were not detected in the brain of (a) SHAM, (b) CHF and (c) normal control mice, while the image in (d) depicts severe Thioflavin S-stained amyloid plaques (indicated by arrows) in the cortex and hippocampal area of APP/PS1 transgenic mice (12 months old). Scale bar: 500 µm.
(TIF)
