# Supplementary figures and images for: Increases in the Risk of Cognitive Impairment and Alterations of Cerebral β-amyloid Metabolism in Mouse Model of Heart Failure
Source: PLoS One. 2013 May 30;8(5):e63829. doi: 10.1371/journal.pone.0063829 (PMC3667825; doi:10.1371/journal.pone.0063829)

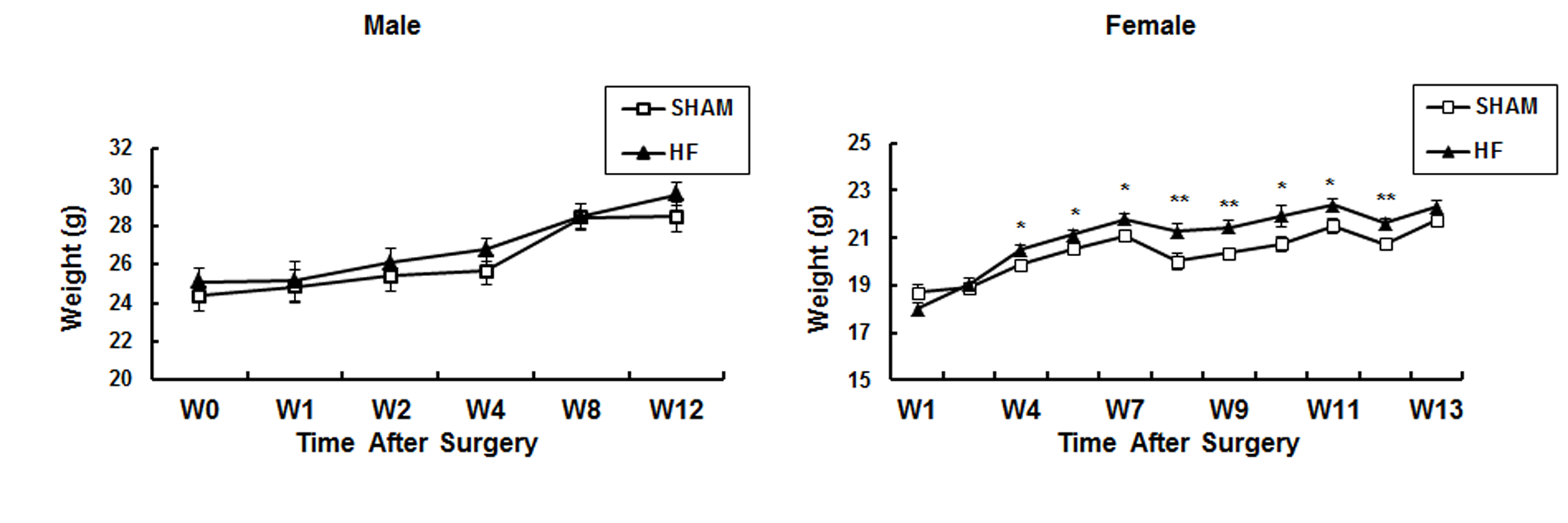

Supplement: Figure S1 — Mouse weight surveyed during the experimental procedures. There was no difference in the gain of weight in male mice of HF and SHAM groups; while the weight of female mice with surgery was significantly higher than that of SHAM group during W4 and W12, *P<0.05 or **P<0.01, n = 8–10 for SHAM and n = 10–12 for HF group. (TIF) [file pone.0063829.s001.tif]

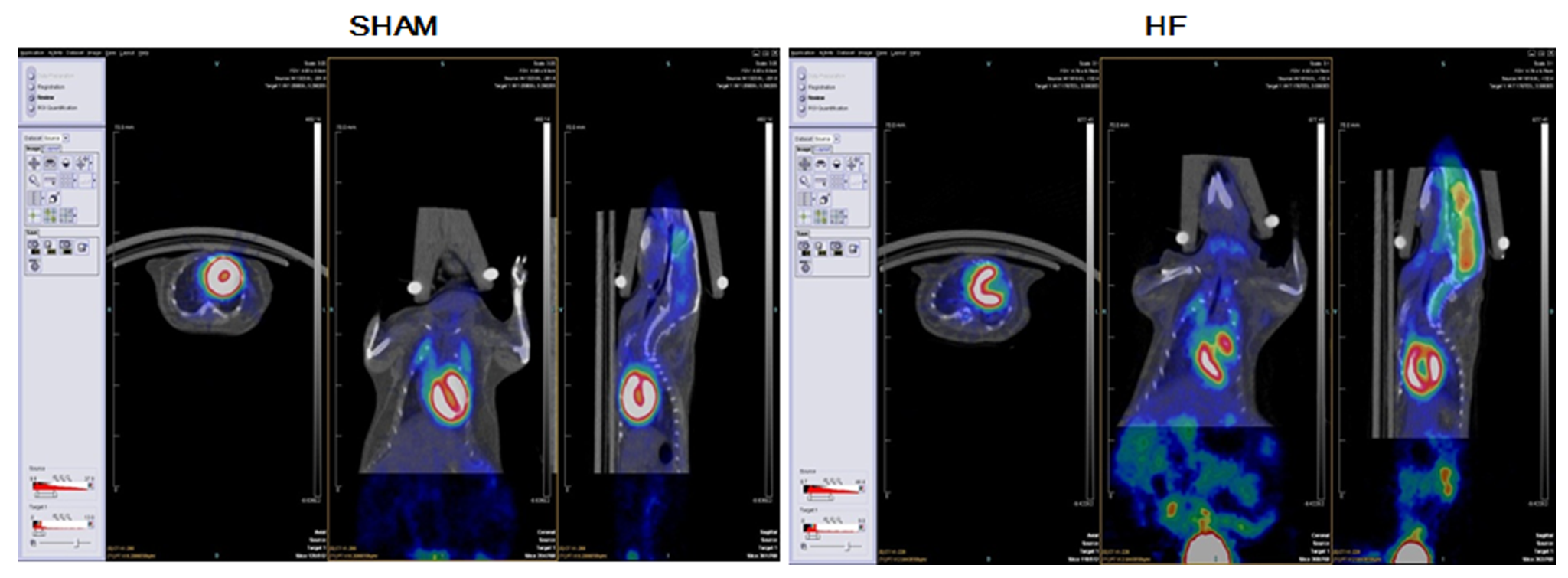

Supplement: Figure S2 — 18F-FDG MicroPET/CT imaging exhibiting the glucose uptake and metabolism in the heart and brain of female SHAM and CHF mice at six months post-surgery. (TIF) [file pone.0063829.s002.tif]

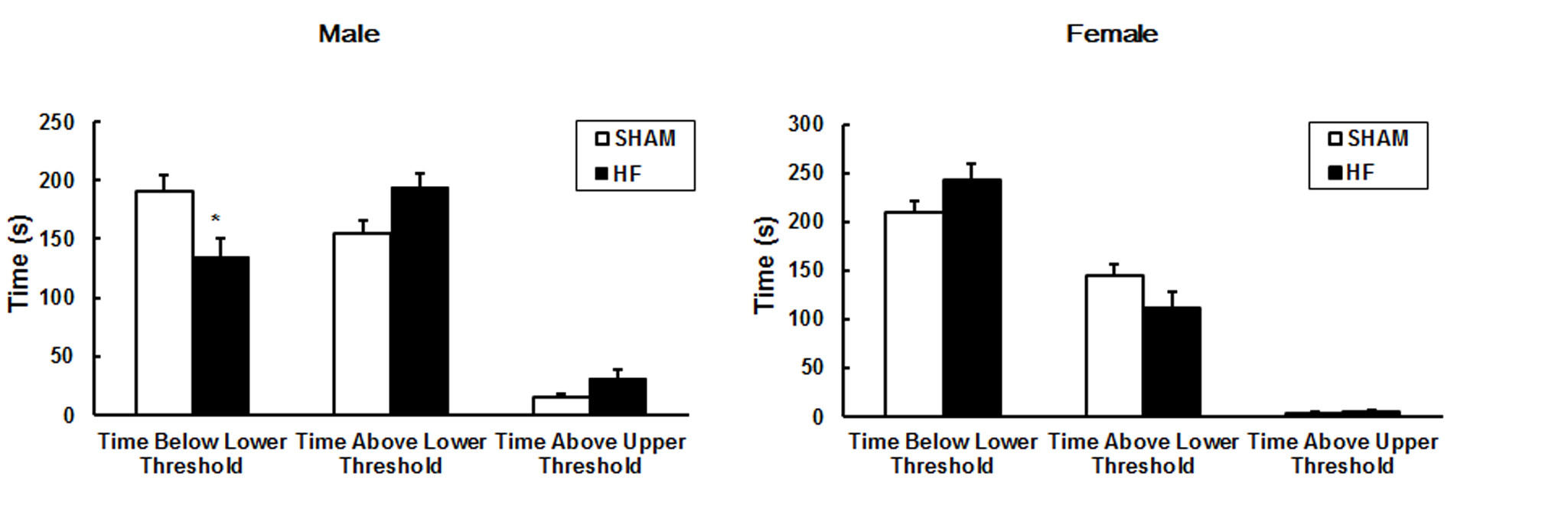

Supplement: Figure S3 — The result from tail suspension test. Tail suspension test was used to assess the depression-like behaviors of CHF and SHAM mice. The time of immobility and struggle was measured during a 6 minutes tail suspension test. Test was performed three months after surgery. Data are expressed as mean ± SE, *P<0.05, n = 5–10 for SHAM and n = 5–11 for CHF group. (TIF) [file pone.0063829.s003.tif]

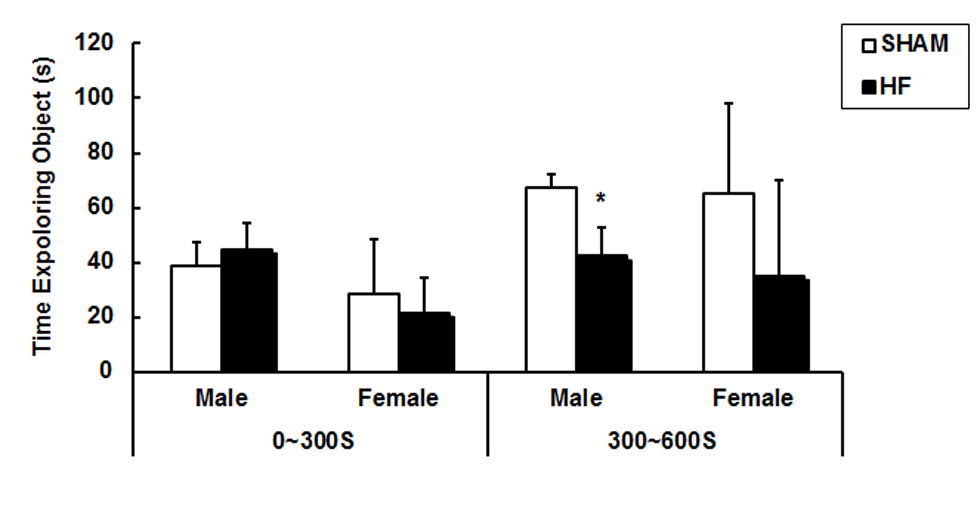

Supplement: Figure S4 — The result from novel object recognition test. The novel object recognition task was used to evaluate the cognition ability in CHF and SHAM mice. Mice were exposed to two copies of an object for 10 minutes and then after 60 minutes they were allowed to explore the familiar object and a novel object again for 10 minutes. The object exploration time during the test phase was recorded. Test was performed three months after surgery. Data are expressed as mean ± SE,* P<0.05, n = 6–7 for SHAM and n = 4–5 for CHF group. (TIF) [file pone.0063829.s004.tif]

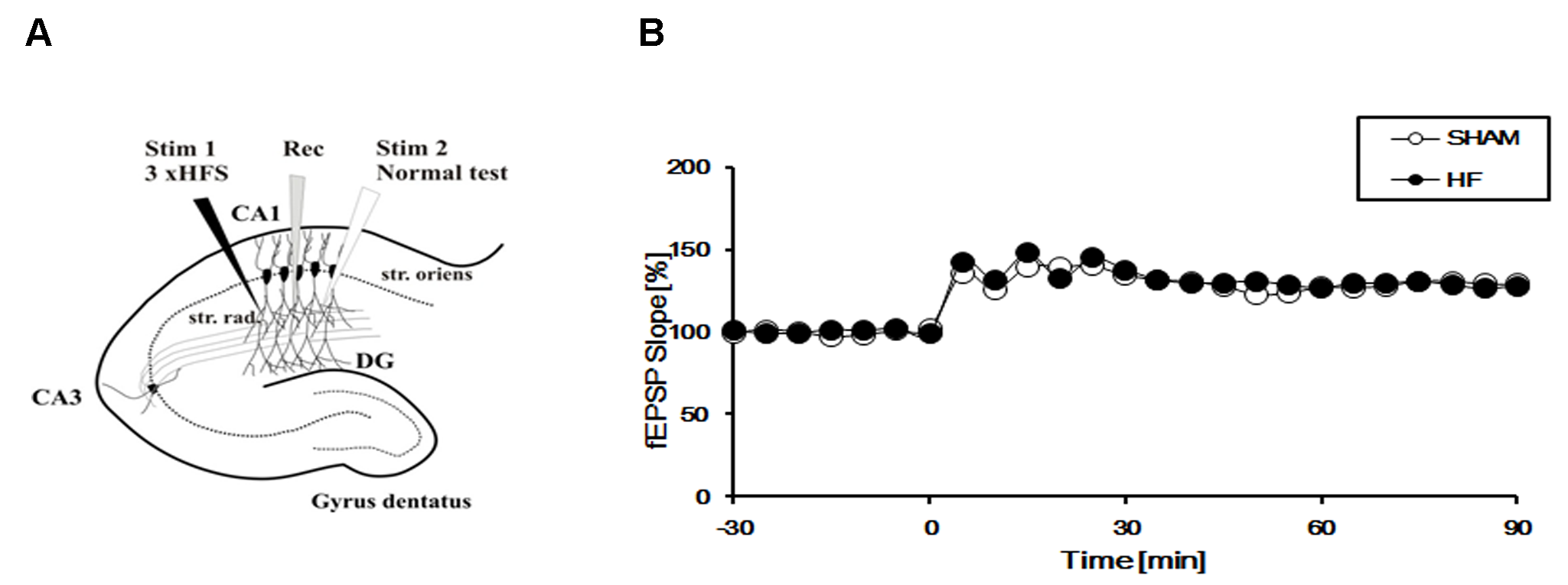

Supplement: Figure S5 — Long term potentiation (LTP) was induced in hippocampal slices of CHF and SHAM mice three months after surgery. (A) Schema of hippocampal slice and position of electrodes. The stimulation electrodes (S1, S2) were placed in the str. rad. of the hippocampal CA1 region facing each other. A recording electrode (gray, Rec) was placed in-between to record evoked fEPSPs. The S2 input was used as a control input to monitor baseline stability. Input S1 was used to evoke synaptic plasticity by high-frequency stimulation (HFS, 100 Hz). (B) LTP was induced by HFS, which consisted of 3 times 100 Hz tetanization (lasting 1 second) with an inter-train interval of 10 minutes. LTP was inducible in the acute slices obtained from CHF and SHAM mice and lasted over 90 minutes. No LTP impairments were detectable in CHF mice. (TIF) [file pone.0063829.s005.tif]

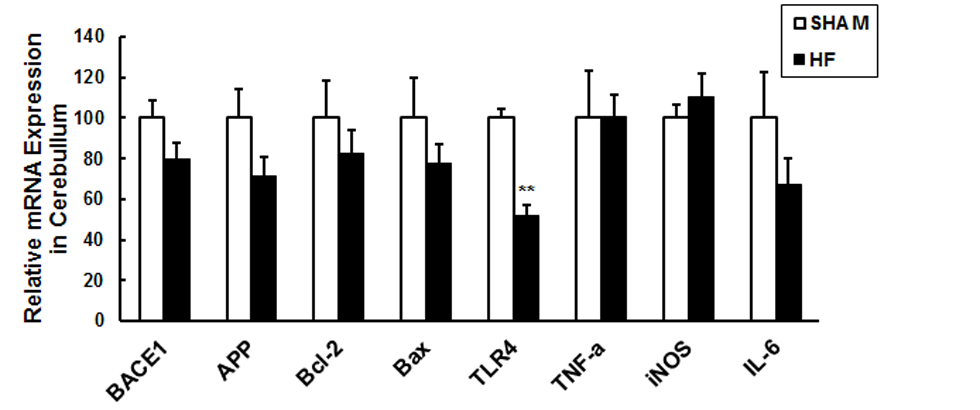

Supplement: Figure S6 — Transcriptional alterations of selected genes detected by real-time PCR in the cerebellum of CHF mice three months after surgery. All mRNA expression levels were normalized to β-actin. Data are presented as mean ± SE, *P<0.05, n = 4–6 for SHAM and CHF group. (TIF) [file pone.0063829.s006.tif]

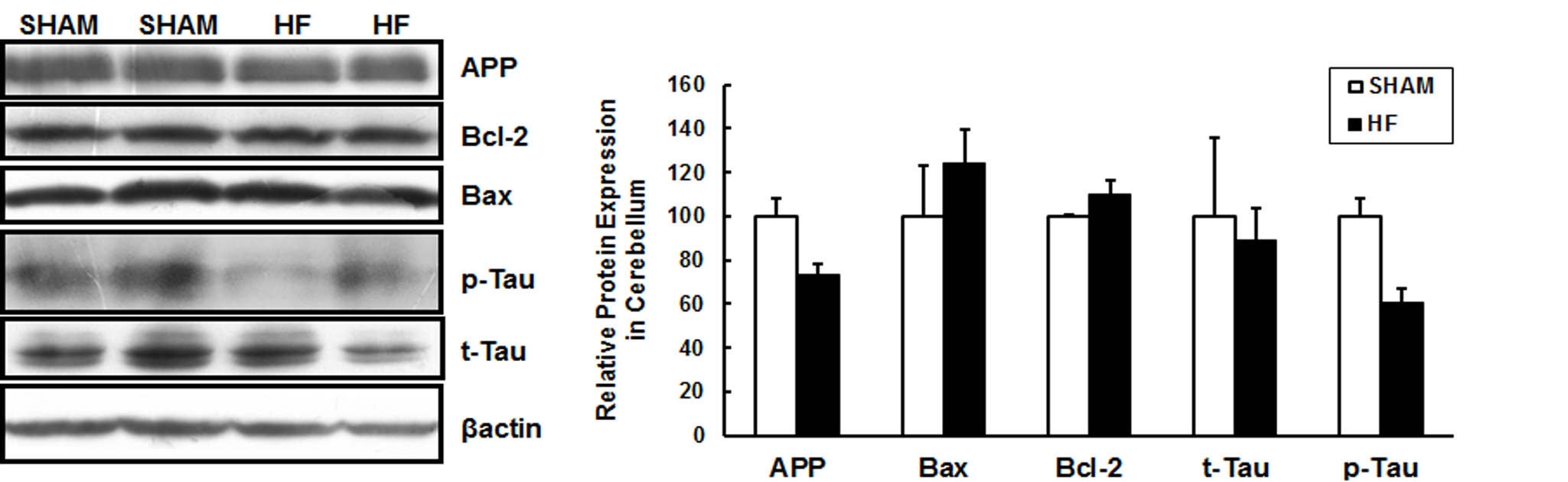

Supplement: Figure S7 — The expressions of APP, Bcl-2, Bax, t-Tau and p-Tau proteins in the cerebellum of SHAM and CHF mice three months after surgery. Representative immunoblots and the summary of the densitometric analysis were shown. Data are presented as mean ± SE, n = 4–6 for SHAM and CHF group. (TIF) [file pone.0063829.s007.tif]

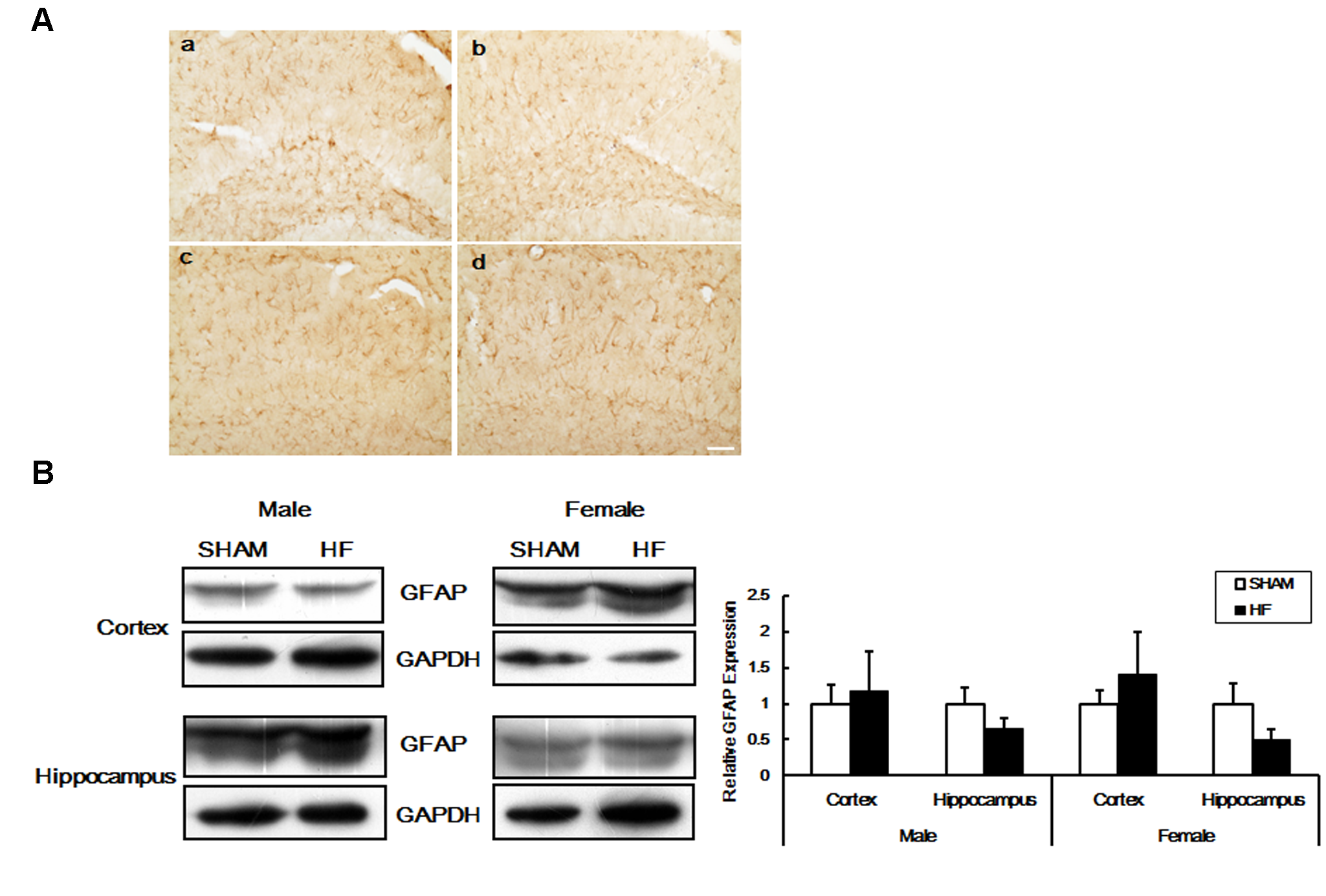

Supplement: Figure S8 — Gliosis was not detectable within the cortex and hippocampus of CHF mice three months after surgery. (A) Immunohistochemistry staining revealed GFAP positive astrocytes within the hippocampus of male (a) SHAM and (b) CHF mice, female (c) SHAM and (d) CHF mice. Scale bar: 20 µm. (B) The levels of GFAP proteins in the cortex and hippocampus of SHAM and CHF mice were determined by Western blot, n = 4–6 for SHAM and CHF group. (TIF) [file pone.0063829.s008.tif]

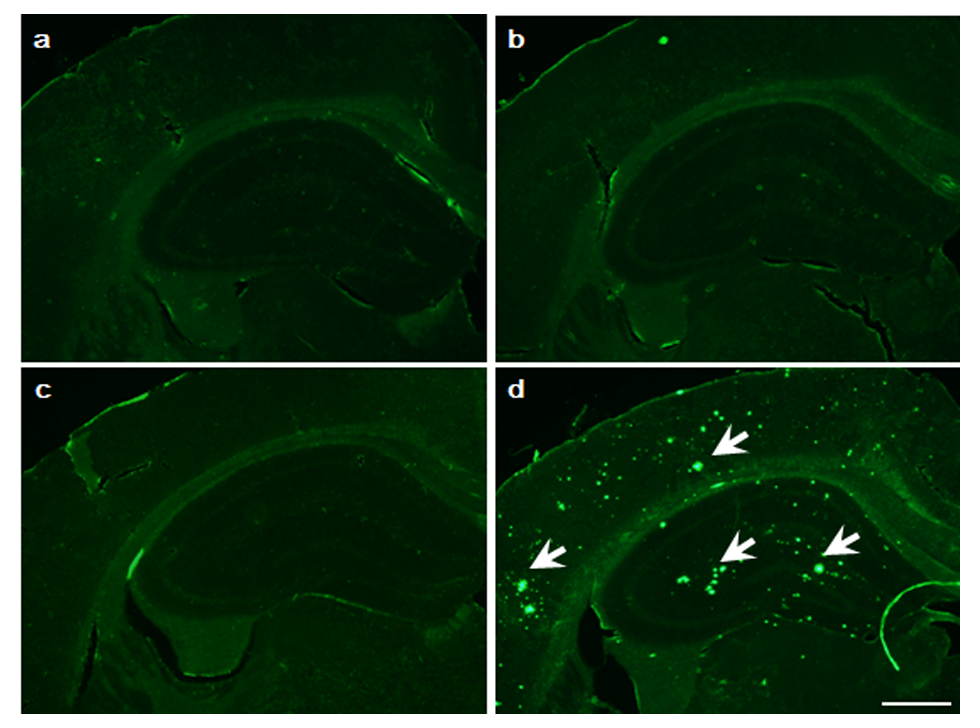

Supplement: Figure S9 — No amyloidogenesis on brain slices of female CHF mice six months after surgery. Brain slices were stained with Thioflavin-S. Thioflavin S-stained amyloid plaques were not detected in the brain of (a) SHAM, (b) CHF and (c) normal control mice, while the image in (d) depicts severe Thioflavin S-stained amyloid plaques (indicated by arrows) in the cortex and hippocampal area of APP/PS1 transgenic mice (12 months old). Scale bar: 500 µm. (TIF) [file pone.0063829.s009.tif]
